# Exercise Interventions as the Primary Treatment for Depression: Evidence from a Narrative Review

**DOI:** 10.21315/mjms2020.27.5.2

**Published:** 2020-10-27

**Authors:** Nur Fatin Nabilah Md Zemberi, Muhammad Mokhzani Ismail, Mohammad Farris Iman Leong Abdullah

**Affiliations:** Lifestyle Science Cluster, Advanced Medical and Dental Institute, Universiti Sains Malaysia, Kepala Batas, Pulau Pinang, Malaysia

**Keywords:** depressive disorders, exercise intervention, aerobic exercise, progressive resistance training, primary treatment of depression

## Abstract

There is an increasing evidence supporting the efficacy of exercise interventions in the treatment of depression, which is a growing global health concern. However, data on the efficacy of exercise as the primary treatment for depression are scarce. This narrative review explored the efficacy of exercise interventions as the primary treatment for depressive disorders. A comprehensive search for English-language literature published between January 1965 and November 2019 was conducted via PubMed, Google Scholar, Scopus, Web of Science, PsycINFO, EMBASE, Cochrane database and Medline. Thirteen randomised control trials (RCTs) were included in the final analysis. Their results indicated that supervised aerobic exercise and high-intensity progressive resistance training (PRT) were effective in ameliorating depressive symptoms as the primary treatment compared with control groups, but they were not superior to other active treatments, such as antidepressants and cognitive behavioural therapy. Aerobic exercise and high-intensity PRT may be a promising primary treatment for depression as they may induce biopsychosocial effects (effects on neurotrophic factor, pro-inflammatory cytokines, monoamine, the hypothalamic-pituitary-adrenal axis, self-efficacy, mastery experience, adaptive coping and social interaction), which may ameliorate the severity of depressive symptoms. However, future RCTs with more comprehensive and well-designed methodologies are warranted to confirm our findings.

## Introduction

Depression has become a major health burden; it is a disorder that currently affects 264 million people worldwide (1). The rapid rise in the incidence of depression is well documented, with the number of cases increasing from 172 million in 1990 to 258 million in 2017 (2). The mainstay of treatment for depression is pharmacotherapy (antidepressants) and psychotherapy (particularly cognitive behavioural therapy [CBT]), but these treatments are costly and associated with adverse effects; moreover, they exhibit high dropout rates (3–5). In addition, depressive disorder is one of the most prevalent psychiatric disorders associated with suicidal behaviour. Hence, depressive disorder should be adequately treated to prevent the increasing trend of suicidal behaviour, which results in a loss of valuable life (6).

As an alternative to conventional treatments, exercise intervention is gaining increasing attention as a treatment option for depression and it is now recommended by clinical guidelines for the treatment of depression (7, 8). Long-term physical exercise has been shown to increase the levels of a number of trophic factors, such as brain-derived neurotrophic factor, neurotrophin-3, epidermal growth factor, vascular endothelial growth factor and glial cell line-derived neurotrophic factor. These factors promote angiogenesis, synaptogenesis and neurogenesis, which in turn lead to neuron survival, proliferation and maturation. Hence, physical exercise may lead to reduced depressive symptoms (9, 10).

Several systematic reviews, meta-analyses and literature studies have pointed out the efficacy of exercise interventions in treating various subtypes of depressive disorders, such as major and minor depression and dysthymia. Such research has mostly considered exercise as an adjunctive therapy to primary treatment with antidepressants or CBT across a variety of age groups, from young adults to the elderly (18–above 65 years old) (11–16). However, few reviews have focussed on providing an overview of the efficacy of exercise interventions as the primary treatment for depressive disorders (i.e. in patients who are not currently being treated with antidepressants and psychotherapy). Hence, the goal of this comprehensive narrative review of the literature was to explore the types of exercise intervention that show efficacy as the primary treatment for depressive disorders and how their efficacy fares relative to other active depression treatments. In this narrative review, we applied the methodological rigour of a systematic review; to accomplish this, we carried out the following steps: we focussed our review based on well-defined objectives; established clear inclusion and exclusion criteria for the electronic literature search; established relevant criteria for literature selection based on the eligibility criteria; and critically assessed the methodology, key results, interpretation of findings and limitations of the selected studies (17).

## Methods

### Search Strategy

An electronic literature search of papers published from January 1965 to November 2019 was performed using the following major databases: PubMed, Google Scholar, Scopus, Web of Science, PsycINFO, EMBASE, Cochrane database and Medline. A preliminary search was performed with Medical Subject Headings (MeSH) terms and keywords, such as ‘exercise intervention’ and ‘depressive disorders’. A refined search was then carried out with additional keywords, such as ‘low intensity exercise’, ‘moderate intensity exercise’, ‘high intensity exercise’, ‘minor depression’, ‘major depressive disorder’, ‘premenstrual dysphoric disorder’, ‘dysthymia’, ‘physical activity for depressive disorder’, ‘exercise intervention for depressive disorder’, ‘aerobic exercise’, ‘stretching exercise’ and ‘home-based exercise’. The search was conducted independently by all three authors, and the search findings were compared; discrepancies were resolved before the final selection of articles to be reviewed.

### Inclusion Criteria

Studies were eligible for review if they met the following criteria: i) they were published in English-language, peer-reviewed journals; articles in-press were included in the search; ii) they involved randomised control trials (RCTs) that investigated the efficacy of any exercise intervention as a stand-alone intervention for treatment of depression; if patients were on an antidepressant, it had been used for < 2 weeks; and iii) they included subjects aged 18 years and above, of any gender, diagnosed with any depressive disorder, and with any severity of depressive symptoms confirmed by screening with validated diagnostic and rating tools for depressive disorders. Studies were excluded if they had the following characteristics: i) they were published in languages other than English; ii) they took the form of systematic reviews, meta-analyses, case reports and series, theses, letters to the editor and editorials or academic conference proceedings; iii) they included subjects diagnosed with mental disorders other than depressive disorders; and iv) they did not give sufficient information about the efficacy of the exercise intervention and diagnoses of the subjects.

## Results

### Search Results

**Figure 1:**
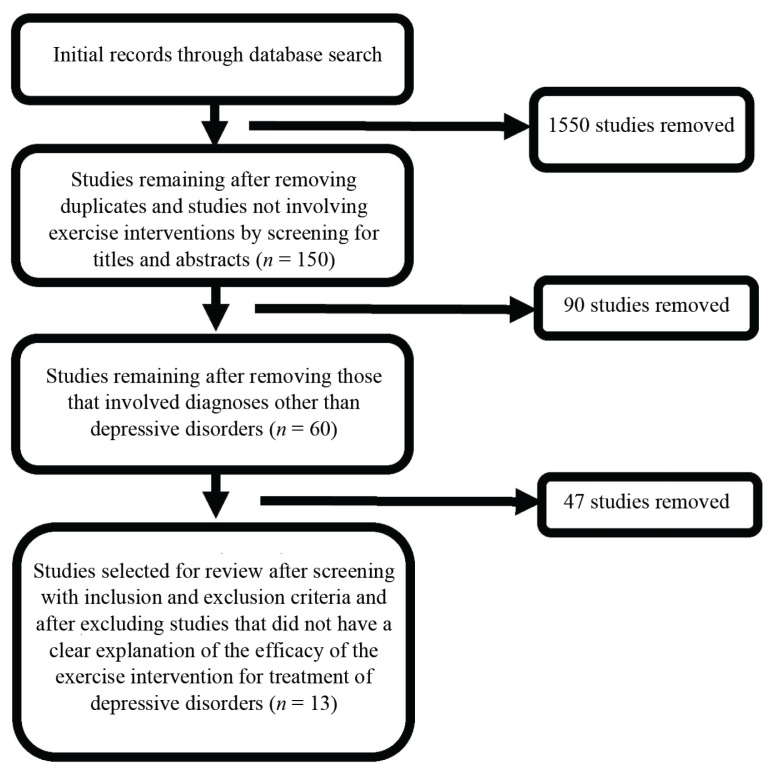
Flowchart of study selection for review

The initial database search of the study titles and abstracts yielded 1700 studies, but 1550 studies were removed as they did not involve exercise intervention or were duplicates. Careful screening of the abstracts of the remaining 150 studies led to the removal of another 90 studies, which included diagnoses of mental illnesses other than depressive disorders. The full-text articles of the remaining 60 studies were screened and 47 were excluded because they did not provide a clear explanation of the efficacy of exercise intervention for the treatment of depressive disorders or they did not clarify if participants were on any treatment for depression other than exercise interventions. Hence, 13 studies were used in this review. The database search and selection of studies are summarised in Figure 1, while Table 1 provides a summary of all the selected studies.

### Study Participants

The total number of participants in the 13 studies reviewed was 1049 patients. The sample sizes ranged from 32–202 patients. Eight studies had relatively small sample sizes, while the other five had relatively large ones (i.e. > 100 patients) (18–22). The participant age ranged from 18–above 60 years old. Six studies recruited participants who were > 60 years old (22–27). The male-to-female ratios ranged from 1:1–1:3 in 11 studies (18–20, 22–27, 29, 30); the other two studies had ratios of 2:1 and 3:1 (21, 28). Eleven studies included outpatients with depression (18–27, 30), one included inpatients (28) and another recruited university students with depression (28).

### Study Characteristics

All 13 studies selected for review conducted an RCT. Table 1 summarises the study characteristics.

## Exercise Interventions

One study employed a two-armed RCT that compared the efficacy of supervised aerobic exercise with a placebo (stretching exercise in which interaction with healthcare providers was similar to that in the intervention group) (20). Another three studies employed two-armed RCTs to compare moderate and high-intensity progressive resistance training (PRT) with a control group (no intervention, health education) (23, 24, 26). A three-armed RCT study compared high-intensity PRT with low-intensity PRT and a control group (standard care from a general practitioner [GP]) (25). In another three-armed RCT, Blumenthal et al. (18) compared supervised aerobic exercise, sertraline (an antidepressant drug) treatment alone and combined supervised aerobic exercise with sertraline treatment without a control group. Brenes et al. (27) used a three-armed RCT approach to compare supervised aerobic exercise with sertraline-only treatment and a control group (usual treatment). Sadeghi et al. (28) conducted a three-armed RCT to compare supervised aerobic exercise with CBT and a control group (no treatment). In another three-armed RCT, supervised aerobic exercise was compared with placebo exercise (stretching exercise) and a control group, and all participants took an antidepressant for < 2 weeks (29). A three-armed RCT compared supervised aerobic exercise with sertraline alone and a placebo group (21). Belvederi Murri et al. (22) carried out a three-armed RCT that compared sertraline plus progressive aerobic exercise with sertraline plus non-progressive exercise and sertraline alone. Dunn et al. (30) conducted an RCT with a 2 × 2 factorial design to compare aerobic exercise at the public health dose (PHD; 17.5 kcal/kg/week, 5 times/week) with aerobic exercise at low dose (LD; 7 kcal/kg/week, 3 times/week) and a control group (no treatment). Finally, an RCT with parallel groups was conducted to compare supervised aerobic exercise with home-based exercise, sertraline-only treatment and a control group (placebo pill titrated to be similar to sertraline) (19).

## Duration of Intervention

The longest duration of the intervention was 24 weeks in one study (22). One study conducted intervention for 20 weeks (24). Four other studies conducted interventions for 16 weeks (18–19, 21, 27). Two studies conducted interventions for 12 weeks or 3 months (20, 30). Four studies conducted interventions for only 10 days–10 weeks (23, 25–26, 29). One study did not specify the duration of the intervention conducted (28).

## Outcome Measures

The severity of depression was assessed using several validated depressive symptom screening tools, such as the Beck Depression Inventory (BDI) (18, 20, 23, 24, 26, 27), 17-item Hamilton Rating Scale for Depression (HAMD-17) (18–23, 25, 27, 30), 6-item HAMD (HAMD-6) (20), and Geriatric Depression Scale (GDS) (25–27). Severity of depressive symptoms assessment was performed at baseline and post-intervention in nine studies (i.e. two timepoints) and eight of the studies repeated the assessment immediately after the intervention (18–21, 23, 25, 29, 30), whereas one study repeated the assessment 8 weeks after completion of the intervention (28). Two studies performed assessments of depressive symptoms at three timepoints (baseline, immediately post-intervention and 4 or 6 months after intervention) (26, 27). One study performed depressive symptoms assessment at baseline; at 6, 10 and 20 weeks from the start of the intervention; and at 26 months after completion of the intervention (24). Finally, one study performed depressive symptoms assessment at baseline and after 4 weeks, 8 weeks, 12 weeks and 24 weeks from the beginning of the intervention (22). Ten studies compared the severity of depressive symptoms between baseline and post-intervention, measured by HAMD-17, BDI, GDS and HAMD-6 as their primary outcomes (18, 20, 21, 24, 26–30). One study measured clinical response (i.e. 50% reduction in HAMD-17 post-intervention) (19) and two studies assessed remission (i.e. HAMD-17 ≤ 10 and HAMD-17 ≤ 8 post-intervention) as their primary outcomes (22, 23).

## Quality of Study Assessment

Regarding the randomisation process, only six studies employed the allocation concealment method (19–21, 24, 25, 30), while another four studies did not (18, 22–23, 29). Two studies did not report on the randomisation method (27, 28) and one did not blind the participants (26). The investigators were blinded in eight of the studies (19–21, 23–25, 27, 30), and three studies did not mention blinding (18, 28, 29). Unequal proportions of participants with major and minor depression were randomised to the intervention and control groups in two studies (23, 25). The proportions of females and males randomised to the intervention and control groups were unequal in seven studies (21–23, 25–27, 30). One study had no dropout participants (23), while attrition rates in another nine studies ranged from 1.5% to 20.5% (18–20, 22, 24–26, 29, 30); three studies did not report the attrition rate (21, 27, 28). Ten studies performed data analysis with the intent-to-treat principle (18–22, 24, 26, 27, 29, 30). All but one study compared exercise interventions with a control or placebo group (18).

## Efficacy of Exercise Intervention Immediately Post-Intervention

Seven studies reported the efficacy of exercise intervention for the treatment of depressive disorders immediately after completion of the intervention (Table 1). Of the four studies that compared supervised aerobic exercise with a control group, three reported that supervised aerobic exercise led to a significantly higher reduction in the severity of depressive symptoms immediately post-intervention compared with that of the control group (19, 29, 30). However, another study reported that supervised aerobic exercise did not lead to a significantly greater reduction in severity of depressive symptoms post-intervention when compared with the control group (stretching exercise) (20). Dunn et al. (30) reported that the PHD of supervised aerobic exercise reduced the severity of depressive symptoms by 47% at the post-intervention measurement compared with the LD of supervised aerobic exercise (30%) and the control group (no exercise, 29%). Blumenthal et al. (19) showed that supervised aerobic exercise led to remission (HAMD ≤ 8) in 45% of participants post-intervention compared with home-based exercise (40% remission) and placebo (31% remission), but the sertraline-only treatment group had the highest remission, at 47% (19).

Blumenthal et al. (21) revealed that supervised aerobic exercise and sertraline reduced the severity of depressive symptoms at 4 months after the beginning of the intervention compared with the administered placebo, but there was no difference in the reduction of severity of depressive symptoms between the supervised aerobic exercise and sertraline groups. Legrand and Neff (29), who recruited all patients with antidepressant treatment of < 2 weeks, indicated that the effect size of depressive symptoms reduction at the post-intervention measurement was significantly greater for supervised aerobic exercise compared with stretching exercise and control groups. In contrast, two studies compared active treatment groups. Blumenthal et al. (18) reported that no significant differences in the reduction of depressive symptoms post-intervention were detected among supervised aerobic exercise, sertraline-only treatment and combined sertraline and supervised aerobic exercise treatment. Belvederi Murri et al. (22) reported a greater remission rate in sertraline plus progressive aerobic exercise and sertraline plus non-progressive exercise groups compared with sertraline-only treatment after 24 weeks of intervention. Shorter time to remission was also observed in the sertraline plus progressive aerobic exercise group compared with the sertraline-only group. Two studies compared high-intensity PRT with a control group in elderly patients immediately post-intervention (23, 25). Singh et al. (23) reported that high-intensity PRT resulted in a significantly greater reduction in depressive symptoms compared with the control group (health education) at the post-intervention measurement, and Singh et al. (25) found that high-intensity PRT caused a significantly greater reduction in depressive symptoms (61% of participants) compared with low-intensity PRT (29%) and the control (standard GP care; 21%) post-intervention.

## Efficacy of Medium- and Long-Duration Exercise Intervention

Two studies investigated the efficacy of PRT compared with control groups in elderly patients (24, 26). Sims et al. (26) reported that moderate-intensity PRT did not result in a significantly greater reduction in depressive symptoms compared with the control group in elderly patients immediately and 6 months after the intervention. Singh et al. (24) demonstrated that high-intensity PRT led to a significantly greater reduction in depressive symptoms compared with controls (health education) in elderly patients at 20 weeks of intervention and 26 months after the intervention. The antidepressant effect was maintained at 20 weeks of intervention and 33% of the participants still performed regular exercise after 26 months without supervision.

One study reported that supervised aerobic exercise and CBT led to a significant reduction of depressive symptoms compared with the control group at 8 weeks after completion of the intervention, but the exercise group did not differ from the psychotherapy group in terms of a reduction in depressive symptoms (28). Finally, another study demonstrated that supervised aerobic exercise with resistance training and sertraline-only treatment groups exhibited significantly greater reduction in the clinician-administered rating tool (HAMD-17) but not in the self-administered tool (GDS) in elderly patients at 4 months post-intervention (27).

## Discussion

### Efficacy of Supervised Aerobic Exercise and High-Intensity Progressive Resistance Training as Primary Treatments for Depression

This comprehensive narrative review examined the efficacy of exercise interventions as the primary treatment for depressive disorders. Four studies indicated that, compared with control groups, supervised aerobic exercise (daily or 3–5 days/week for 30–60 min per session) for 10 days–16 weeks is efficacious for reducing depressive symptoms in patients with major and minor depressive disorders (19, 28–30). Only one study reported that supervised aerobic exercise was no better than stretching exercise (control group) in reducing depressive symptoms (20). However, only Blumenthal et al. (19) recruited a relatively large sample (*n* > 100) compared with the other three studies (sample sizes = 35 to 80) (28–30). Other study limitations of note were as follows: i) Blumenthal et al. (19): most of the participants had only mild depression, and the placebo response rate was high; ii) Krogh et al. (20): the study stopped before achieving the pre-planned sample size; iii) Sadeghi et al. (28): randomisation concealment, blinding and attrition rates were not mentioned, and data analysis was not performed according to the intent-to-treat principle; and iv) Legrand and Neff (29): blinding and medication dosage were not mentioned, and the intervention was of relatively short duration. The efficacy of supervised aerobic exercise was not superior to that of other active treatments, such as antidepressants and CBT (18, 19, 21, 26). However, Blumenthal et al.’s (18) study was limited by the lack of a control group and no mention of blinding. Belvederi Murri et al. (22) indicated that a combination of supervised aerobic exercise (progressive or non-progressive) with antidepressant (sertraline) had superior efficacy compared with sertraline-only treatment in ameliorating depressive symptoms and inducing remission. Yet, the main limitation of this study was that it included only elderly major depressive patients (age 65–85 years), and hence, the results cannot be generalised to younger adult depressed patients.

Three studies demonstrated that, when compared to control groups, high-intensity PRT (3 days/week for 40–60 min per session) for 8 weeks–26 months was efficacious for reducing depressive symptoms in patients with major and minor depressive disorders and dysthymia (23–25). However, these studies only recruited elderly patients (> 60 years old) and had relatively small sample sizes (*n* = 32–60). Singh et al. (25) also had a short duration of intervention (only 8 weeks). In contrast, Sims et al. (26) reported that moderate-intensity PRT was not efficacious for reducing depressive symptoms in patients with unspecified depression compared with controls. However, Sims et al. (26) recruited only 36 participants, the duration of the intervention was short (10 weeks) and participants were not blinded. Only one study reported the efficacy of combined supervised aerobic exercise with resistance training (3 days/week, 60 min per session) for 16 weeks; they found reduced depressive symptoms in elderly patients (≥ 65 years old) with minor depression as compared to the control group. However, the sample size was small and the attrition rate was not mentioned (27).

It was difficult to compare studies because the selected studies showed heterogeneity in terms of sample size, sample characteristics, methodology, randomisation, blinding strategies, modes of intervention, duration of intervention, nature of the control groups, unequal distribution of gender between groups and different outcome measures applied. However, from our findings, we can conclude that supervised aerobic exercise and high-intensity PRT may be efficacious as a primary treatment for depressive disorders, even without the prescription of antidepressants. Nonetheless, we recommend that more multicentre double-blinded RCTs with larger sample sizes, equal gender distributions across comparison groups and longer follow-up durations with a higher number of repeated assessments be conducted to confirm the efficacy of supervised aerobic exercise as a primary treatment for depressive disorders. In addition, multicentre double-blinded RCTs with larger sample sizes and equal gender distributions across comparison groups, as well as involving patients from a younger age group, are warranted to confirm the efficacy of high-intensity PRT as a primary treatment for depressive disorders. Furthermore, future studies should evaluate the efficacy of exercise intervention conducted in individuals as all the studies reported herein conducted exercise intervention in groups, and therefore, they could not control for the confounding effect of social interactions and social support in the groups, which may themselves improve depressive symptoms.

### Biopsychosocial Model of the Mechanisms Underlying the Efficacy of Supervised Aerobic Exercise and Progressive Resistance Training as Primary Treatments for Depression

**Figure 2:**
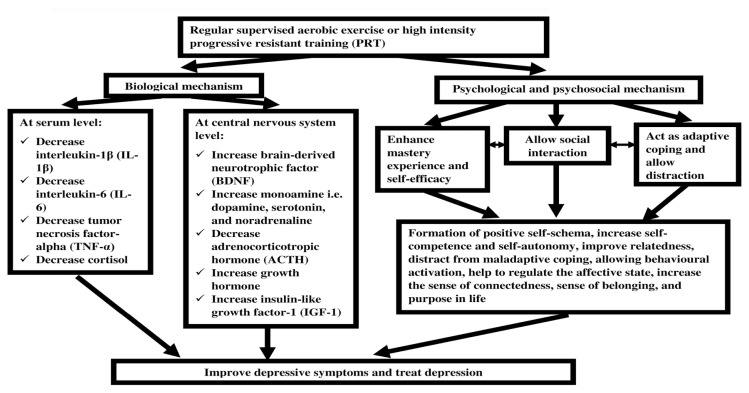
The biopsychosocial mechanism underlying the effect of exercise on depression

The mechanism behind the efficacy of supervised aerobic exercise and PRT as a primary treatment for depression can be explained in terms of the biopsychosocial model. The biopsychosocial mechanism underlying the effect of exercise on depression is illustrated in Figure 2.

## Biological Mechanism Underlying the Effect of Exercise on Depression

One important neurotrophic factor in the brain that exercise interacts with is brain-derived neurotrophic factor (BDNF), which is involved in the regulation of neuroregeneration, neurogenesis, neuroprotection and synaptic plasticity (31). While BDNF decreases in depression, exercise tends to increase the level of BDNF in the brain, which may bring about an amelioration of depressive symptoms similar to the effects of antidepressant treatment (32). Exercise stimulates the release of various proteins from the neurons in the brain, and one of these is BDNF (33); BDNF may produce an antidepressant effect by stimulating neurogenesis and having a neuroprotective influence on the hippocampus (34), a vital brain structure that is involved in the regulation of emotional memory (35). An increase in hippocampal volume and size has been observed in subjects performing aerobic exercise for duration of 3 months–1 year (36, 37). In a randomised clinical trial of patients with depression, subjects were randomised to an aerobic exercise plus treatment-as-usual group and treatment-as-usual only group, where treatment as usual included antidepressant treatment and psychotherapy; as a result, a significant increase in serum BDNF was observed in the exercise plus treatment-as-usual group compared with the treatment-as-usual only group (34).

Pro-inflammatory cytokines have been reported to induce depressive symptoms when their serum level increases. Various pro-inflammatory cytokines are increased in the serum of depressed patients compared with non-depressed patients, including interleukin-1β (IL-1β), IL-6 and tumour necrosis factor-α (TNF-α) (38). Again, aerobic exercise for a duration of 3 months has been shown to significantly reduce serum IL-1β, IL-6 and TNF-α, which is also positively correlated with an improvement in depressive symptoms in depressed subjects (39, 40). When aerobic exercise intervention is compared with other exercise, such as strength exercise, there is a significantly greater decrease in pro-inflammatory cytokines, including TNF-α and C-reactive protein (41).

Dysregulation in the hypothalamic-pituitary-adrenal (HPA) axis is thought to be a risk factor for developing depression, in which the serum cortisol level is increased and there is blunting of the dexamethasone suppression test in depressive patients (42). The effect of regular exercise on the HPA axis is still inconclusive. The basal level of serum cortisol has been reported to increase in response to regular and chronic exercise (43), but one study indicated that regular exercise significantly reduces urinary cortisol levels, serum adrenocorticotropic hormone (ACTH) and depressive symptoms (44). Other hormonal systems may also play a role in the mechanism underlying the effect of exercise on depression, such as the involvement of growth hormone and insulin-like growth factor-1 (IGF-1), which are associated with neuroprotective effects and regulate mood and sleep in the central nervous system (45, 46). Growth hormone is found to increase in major depressive disorder patients after acute episodes of aerobic exercise and PRT (47). Although IGF-1 has been reported to exert an antidepressant effect in animal studies (48), regular aerobic exercise in depressed human subjects does not result in an increased serum IGF-1 level (49). Hence, the role of IGF-1 in the mechanism underlying the therapeutic effect of exercise on depression is inconclusive.

Regular exercise may alter monoamine levels in the brain. The effect of aerobic exercise on serotonin differs between brain regions and is time-course dependent. Upon acute aerobic exercise, the serotonin level increases in the mid-brain, striatum, hippocampus, cerebral cortex and brainstem. The cortical changes last for a week after discontinuation of exercise, while serotonin metabolism in the hippocampus reduces 1 day after acute exercise but increases again after 1 week of exercise cessation. In terms of dopamine, upon aerobic exercise, the dopamine level is increased in the hippocampus, striatum, mid-brain and brainstem, modulating the mood and memory (50). Similarly, chronic treadmill and wheel exercise in animal studies are shown to increase noradrenaline levels in the pons, hippocampus and amygdala (51). According to the monoamine hypothesis of depression, an increase in serum serotonin, dopamine and noradrenaline will ameliorate depressive symptoms in patients.

## Psychological Mechanism Underlying the Effect of Exercise on Depression

There are several psychological mechanisms underlying the effect of exercise on depression. Exercise can alleviate stress and tends to improve self-efficacy, self-confidence, self-perception and body image, while it prevents negative thoughts. Exercise also improves satisfaction with life and well-being. Regular exercise improves self-efficacy by increasing one’s belief in one’s ability to accomplish tasks and reach a certain level of achievement. This is accomplished by allowing oneself to gain mastery experiences via performing the physical activity or watching others, and in turn, allowing oneself to experience a positive affective state during exercise. Improved self-efficacy leads to the formation of a positive self-identity and self-perception as someone who could cope with a negative mood state by accomplishing tasks and reaching certain goals. Eventually, a positive self-schema is formed where one allows oneself to express positive ideas about oneself, generating improved self-esteem. These changes will help to alleviate stress and depressive symptoms (52, 53).

To improve the sense of well-being, it is necessary to ensure the need for self-competence, self-autonomy and improved relatedness. Regular exercise allows one to fulfil the need for self-competence by practicing and improving in the sport or physical activity of choice, improving self-autonomy through mastery experience gained in practicing physical activity and improving relatedness through sharing experience with others by joining group activities. Hence, improving the sense of well-being by fulfilling these psychological needs will reduce depressive symptoms (54).

Regular exercise could also be viewed as a coping mechanism to overpower maladaptive coping in depression, including social isolation and inactivity. Regular exercise contributes to an increase in pleasure and self-achievement, serving as an activity that may distract depressed persons from maladaptive coping, allow behavioural activation and help regulate the affective state (53, 55). Hence, with regular exercise acting as an adaptive coping strategy, it helps to manage stress and depressive symptoms.

## Psychosocial Mechanism Underlying the Effect of Exercise on Depression

By engaging in regular exercise in a group, one can experience a sense of meaning in life when performing the physical activity and sense of closeness with group members and facilitators by sharing experiences that increase the sense of connectedness, sense of belonging and purpose in life. These factors will allow the person to eliminate maladaptive coping like social isolation, and hence, reduce depressive symptoms. Therefore, the social setting of the group physical activity could bring about mental wellness (56–59).

### Limitations

A few limitations of this review should be noted. First, the review only included articles published in English and there may be articles published in other languages that would have fulfilled the eligibility criteria. However, we did not have funding to include experts who could help interpret findings from non-English articles. Studies in published theses were also excluded, yet they could be a valuable source of data. This omission was due to the time constraints that we faced. Nevertheless, we think this review covered most of the literature evaluating the efficacy of exercise interventions as a primary treatment for depression. Second, due to the heterogeneity of the selected studies, a meta-analysis would have been a more ideal way to assess the literature focussed on the efficacy of exercise interventions for treating depression.

## Conclusion

This narrative review included a comprehensive review of the literature that investigated the efficacy of exercise intervention as the primary treatment of depressive disorders, filling the research gap associated with the lack of such data. We found that supervised aerobic exercise and high-intensity PRT may be efficacious in treating depressive disorders, even without the use of antidepressants. Hence, supervised aerobic exercise at a duration of 30–60 min per session, three to five times a week for 12–16 weeks is recommended for adult depressed patients, while high-intensity PRT at a duration of 40–60 min per session, three times a week for 10–16 weeks is recommended for the elderly. Early morning is suggested for exercise, as it is easier to commit in the morning, before the daily routine intrudes. Furthermore, since exercise can help distract from maladaptive coping, promote behavioural activation and help regulate the affective state, it would be ideal to perform exercise in the morning, especially for depressed patients with morning worsening of depressive symptoms. However, the exercise interventions reviewed were not superior to antidepressants and CBT in the treatment of depression. Future RCTs with more comprehensive and well-designed methodologies are warranted to confirm our findings. Studies that investigate the potential mechanism for exercise as the primary treatment of depression are also warranted. Our review provides useful data for clinicians about the need to include exercise interventions for the treatment of depressive patients because such interventions improve both physical outcomes and patients’ mental health.

## Figures and Tables

**Table 1:** Summary of the studies selected for review

Reference	Study design	Participants	Eligibility criteria	Randomisation and blinding	Intervention	Outcome measures	Results	Limitations
Singh et al. (23)	RCT, two-armed	*n* = 32, 62% females, 38% males	Age ≥ 60 years old, diagnosed with major and minor depression, and dysthymia, not currently on depression treatment	Randomisation by computer-generated list in blocks of five. The responders and assessors were blinded	High-intensity progressive resistance training (45–50 min/session, 3 days/week)-supervised (*n* = 17) for 10 weeks versus controls (on health education only) (*n* = 15)	BDI, HAMD-17, PGMS and SF-36 at baseline and post-intervention	Significant reduction in BDI score (*P* = 0.002), HAMD-17 score (*P* = 0.008) and significantly improved bodily pain (*P* = 0.001), vitality (*P* = 0.002), social functioning (*P* = 0.008) and role emotional (*P* = 0.02) subscales of SF-36 in exercise group as compared to control group from baseline to post-intervention. PGMS score also significantly improved in the exercise group as compared to controls at post-intervention (*P* < 0.001).	Small sample size. Included only those ≥ 60 years.
Blumenthal et al. (18)	RCT, three-armed	*n* = 156, 72% females, 28% males	Diagnosed with major depressive disorder with HAMD-17 ≥ 13, not currently on depression treatment. Age ≥ 50 years old.	Randomisation with stratification by severity of depressive symptoms. Blinding not explained	Supervised aerobic exercise (45 min/session, 3 times/week (*n* = 53) for 16 weeks vs sertraline only group (*n* = 48) versus combined exercise and medication group (*n* = 55)	BDI, HAMD-17, STAI, RSES, LSI, and DAS at baseline and post-intervention	No significant differences in the reduction of HAMD-17 and BDI at post intervention between all three groups, although all three groups exhibited significant reduction in HAMD-17 and BDI scores from baseline to post-intervention. Those on medication exhibited the fastest initial response. There were also no significant difference in changes of STAI, RSES, LSI and DAS scores between all three groups at post-intervention.	Blinding not mentioned. Included only those ≥ 50 years. Absence of no-treatment control group
Singh et al. (24)	RCT. two-armed	*n* = 32, 63% females, 37% males	Age > 60 years old, diagnosed with major and minor depression, and dysthymia, not on antidepressant treatment for the last 3 months.	Computer-generated random list concealed in sealed envelopes. The assessors were blinded	Supervised high intensity progressive resistance training for 10 weeks (3 days/week), then unsupervised exercise at home, lab or health club setting for weeks 11–20, then no study requirement and contact with investigators ranged from 22–35 months (*n* = 17) versus controls with health education for 10 weeks, followed by weekly phone calls by investigators for weeks 11–20, then no study requirement and contact with investigators ranged from 22–35 months (*n* = 15)	BDI and PGMS at baseline, 6, 10, 20 weeks of intervention and at 26 months post-intervention	Significant reductions of BDI scores at both 20 weeks and 26 months of follow up in exercise group compared to control group (*P* < 0.05–0.001). At 26-months of follow up, 33% of exercisers still on regular exercise compared to 0% controls (*P* < 0.05). Unsupervised exercise participants maintained antidepressant effect at 20 weeks of intervention	Small sample size. Included only those > 60 years old.
Singh et al. (25)	RCT, three-armed	*n* = 60, 55% females, 45% males	Age > 60 years old, diagnosed with major and minor depression, and dysthymia, not on antidepressant treatment for last 3 months	Computer-generated random number permutation programme in blocks of 15. Randomisation list in opaque sealed envelope. The assessors blinded.	Supervised high intensity progressive resistance training programme (60 min/session, 3 days/week) (*n* = 20) for 8 weeks versus low intensity progressive resistance training programme (60 min/session, 3 days/week) (*n* = 20) versus control group (standard GP care only) (*n* = 20)	HAMD-17, and GDS at baseline and post-intervention. Clinical response defined as 50% reduction in HAMD-17 score at post-intervention	Significant higher proportion of participants with 50% reduction in HAMD-17 observed in high intensity progressive resistant training (PRT) group compared to the other two groups (High PRT = 61% of participants, low PRT = 29% and controls = 21%, *P* = 0.013). Reduction of GDS scores also significantly higher in High intensity PRT group compared to the other two groups at post-intervention (*P* = 0.001).	Small sample size. Short duration of intervention
Dunn et al. (30)	RCTl (2 × 2 factorial design)	*n* = 80, 75% females, 25% males	Age 20–45 years old, diagnosed with mild to moderate major depressive disorder, not on other depression treatment	Sequential opaque in sealed envelope. The assessors blinded.	Aerobic exercise: PHD (17.5 kcal/kg/week, 5x/week) versus LD (7 kcal/kg/week, 3x/week) (*n* = 18) versus controls (no exercise) (*n* = 13) for 12 weeks	HAMD-17 score at baseline and post-intervention	Significant reduction in HAMD-17 score at 12 weeks i.e. reduced 47% from baseline for public health dose (PHD) group as compared to 30% for LD group and 29% for control group. PHD is efficacious to treat mild to moderate depression. LD did not differ from controls.	Small sample size.
Sims et al. (26)	RCT, two-armed	*n* = 38, 66% females, 34% males	Age ≥ 65 years old, depression with GDS score ≥ 11, not currently on treatment for depression	Randomisation conducted by independent person. Only assessors blinded. Participants not blinded	Moderate intensity progressive resistant training (3 times/week) (*n* = 14) for 10 weeks versus control group (no intervention) (*n* = 18)	GDS, CES-D, HAP, PGMS, WHOQOL-BREF, and SEDBS at baseline, 10 weeks and 6 months. Primary outcome was GDS score reduction	No significant difference in the reduction of the GDS scores between exercise and control groups at follow up. At 6 months follow up, lowering of GDS scores between exercise and control groups also showed no significant difference (*P* = 0.08).	Small sample size. Participants not blinded. Short duration of intervention
Blumenthal et al. (19)	RCT (parallel group, placebo control trial)	*n* = 202, 76% males, 24% females	Age ≥ 40 years old, diagnosed with major depressive disorder with BDI ≥ 12, not currently on depression treatment	Computer-generated conditional randomisation (stratified by age, gender and depression severity) with note in sealed envelope. The assessors were blinded	Supervised aerobic exercise (3x/week) (*n* = 51) versus home-based exercise (3x/week) (*n* = 53) versus sertraline only treatment (50–200 mg/day) (n=49) versus placebo (same titration as sertraline (n = 49) for 16 weeks	HAMD-17 score at baseline and post-intervention. Primary outcome was remission (HAMD-17 score ≤ 8)	Remission achieved in 45% of supervised exercise group, 40% of home-based exercise group, 47% of sertraline group, and 31% of placebo group; the differences were not significant (*P* = 0.057). The lowering of HAMD-17 score not significant between all active treatment groups and placebo (*P* = 0.23).	Most of the participants only had mild depression. High placebo response rate
Brenes et al. (27)	RCT, three-armed	*n* = 37, 62% females, 38% males	Age ≥ 65 years old, diagnosed with minor depression with 2–4 symptoms in DSM-IV, not currently on depression treatment	Randomisation by computer-generated list. Concealment not mentioned. The assessors blinded.	Supervised aerobic and resistance training (60 min/session, 3 days/week) (*n* = 14) for 16 weeks versus sertraline only group (*n* = 11) versus control group (usual care) (*n* = 12)	HAMD-17, GDS, and mental health scale of the SF-36 at baseline, post-intervention, and 4 months	Significant reduction in the HAMD-17 scores in the exercise and the sertraline groups as compared to controls which showed a slight increase in the HAMD-17 score at 4 months of intervention (*P* = 0.005). No significant difference in reduction of GDS score between the exercise, sertraline and control groups at 4 months of intervention.	Small sample size. Attrition rate not mentioned
Krogh et al. (20)	RCT, two-armed	*n* = 115, 67% females, 33% males	Age 18–60 years old, diagnosed with major depressive disorder, currently not on depression treatment	Randomisation by stratification by severity of depression and blood pressure. Group assigned by randomisation sequence unknown to the investigators. The outcome assessors were blinded	Supervised aerobic exercise (30 min, 3x/week) (*n* = 56) for 3 months versus stretching exercise as attention control with similar social interaction with health care providers (*n* = 59)	HAMD-17, BDI-II, HAMD-6, WHO-5, and HAM-A-14 at baseline and post-intervention	No significant differences in the reduction of HAMD-17, BDI-II, HAMD-6, HAM-A-14 and WHO-5 scores between supervised aerobic exercise and stretching exercise control groups at post-intervention. Supervised aerobic exercise was not more superior than stretching exercise to treat depression.	Failed to achieve pre-plan number of participants
Blumenthal et al. (21)	RCT, three-armed, placebo control	*n* = 101, 32% females, 68% males	Age ≥ 35 years with documented coronary heart disease and BDI-II score ≥ 7, currently not on antidepressant and psychotherapy	Computer generated randomisation stratified by age, CHD status, and depression severity. Subjects provided numbers in sealed envelope. Assessor blinded.	Supervised aerobic exercise (30 min, 3×/week) (*n* = 37) for 16 weeks versus sertraline only group (*n* = 40) versus placebo group (*n* = 24)	DSM-IV criteria for diagnosis of major depressive disorder, HAMD-17 at baseline and post-intervention. Other measures: heart rate variability, endothelial function assessed by flow mediated dilation, baroreflex sensitivity, and inflammation and platelet function.	Significant reduction in the HAMD-17 scores in the exercise and the sertraline groups as compared to placebo group at 4 months of intervention (*P* = 0.034). No significant difference between exercise and sertraline groups at 4 months of intervention.	Attrition rate not mentioned.
Belvederi Murri et al. (22)	RCT, three-armed	*n* = 121, 71% females, 29% males	Age 65–85 years, diagnosed with major depressive disorder with HAMD-17 ≥ 18, not currently on antidepressant	Computerised randomised permutation blocks method for randomisation by stratification of age, gender, study centre, severity of depression, and past treatments with anti-depressant. Accessor blinded	Sertraline plus progressive aerobic exercise (60 min, 3x/week) (*n* = 42) for 24 weeks versus sertraline plus non-progressive exercise (60 min, 3x/week) (*n* = 37) versus sertraline only group (low dose at 50 mg for 2 weeks) (*n* = 42)	Diagnosis made with MINI, HAMD-17 at baseline and after 4 weeks, 8 weeks, 12 weeks and 24 weeks of intervention. Primary outcome: remission (HAMD-17 ≤ 10). Secondary outcome: changes in severity of depression (continuous HRSD scores)	81% of sertraline plus progressive aerobic exercise group, 73% of sertraline plus non-progressive exercise group, and 45% of sertraline only group achieved remission at 24 weeks of intervention (*P* = 0.001). Shorter time to remission was observed in the sertraline plus progressive aerobic exercise group compared to sertraline only group.	Include only patients with 65–85 years old. No control group.
Legrand and Neff (29)	RCT, three-armed, placebo control	*n* = 35, 69% females, 31% males, all in-patients	Diagnosed with major depressive disorder with BDI-II score ≥ 29, and antidepressant treatment < 2 weeks. Mean age ranged from 41.8–49.1 years	Computer-generated randomisation numbers to assign groups. No stratification. Blinding not mentioned	Supervised aerobic exercise (for 30 min/day) (*n* = 14) versus stretching exercise as placebo (for 30 min/day) (*n* = 11) versus control group (only on antidepressants) (*n* = 10) for 10 days prior to discharge. All participants on antidepressants	BDI-II at baseline and post-intervention	Significant decreased in BDI-II scores post-intervention for both aerobic and stretching exercise groups. The effect size of BDI-II reduction at post-intervention was larger for aerobic exercise group compared to stretching exercise (Cohen’s d = −1.06) and control groups (Cohen’s d = −1.39). Effect size of stretching exercise compared to control was nearly zero (Cohen’s d = −0.33).	Short duration of intervention. Blinding not mentioned. Medication dosage not mentioned
Sadeghi et al. (28)	RCT, three-armed	*n* = 46, 78% males, 22% females, all university students	Diagnosed with depression with BDI-II score of 13–28 (mild to moderate depression), not on treatment. Mean age ranged from 20.92–21.12 years	Participants randomised into three groups. But randomisation procedure not explained. Blinding also not mentioned.	Supervised aerobic exercise (40–50 min/session, duration not mentioned) (*n* = 16) versus CBT (2 sessions/week for first half and 1 session/week for second half, for 12 sessions) (*n* = 16) versus control group (not on any intervention) (*n* = 12)	BDI-II, DAS, and ATQ at baseline and 8 weeks after intervention	Significant reduction in BDI-II, belief in automatic negative thoughts in ATQ, and DAS scores in the CBT group compared to control group (*P* < 0.05) after intervention. Significant reduction only in BDI-II score noted in exercise group as compared to controls. The difference in reduction of BDI-II scores between CBT and exercise groups was not significant at post-intervention	Small sample size. Randomisation and blinding not explained

Notes: PHD = public health dose of exercise, LD = low dose of exercise, HAMD-17 = 17-item Hamilton Rating Scale for Depression, GDS = geriatric depression scale, PGMS = Philadelphia Geriatric Morale Scale, SF-36 = 36-item medical outcomes survey short form, BDI = Beck Depression Inventory, PGMS = Philadelphia Geriatric Morale Scale, CES-D = Center of Epidemiological Study-Depression scale, HAP = Human Activities Profile, WHOQOL-BREF = World Health Organization Quality of Life-BREF scale, SEDBS = Self Efficacy and the Decision Balance Scale, STAI = State-Trait Anxiety Inventory, RSES = Rosenberg Self-Esteem Scale, LSI = Life Satisfaction Index, DAS = Dysfunctional Attitude Scale, BDI-II = Beck Depression Inventory-II, HAMD-6 = 6-item Hamilton Rating Scale for Depression, WHO-5 = World Health Organization Well-being Index, HAM-A-14 = 14-item Hamilton Rating Scale for Anxiety, ATQ = Automatic Thoughts Questionnaire, CBT = cognitive behavioural therapy, DSM-IV= Diagnostic and Statistical Manual for Mental Disorders 4th Edition, MINI= Mini International Neuropsychiatric Interview
